# Evaluation of Enamel Matrix Derivative (EMD) Teratogenicity on the Rat Embryo Neural Crest Culture

**Published:** 2011

**Authors:** Maliheh Mamashli, Mina Ramezani, Maliheh Parsa, Seyyed Nasser Ostad

**Affiliations:** a *Department of Toxicology and Pharmacology, Faculty of Pharmacy and Pharmaceutical Sciences Research Center, Tehran University of Medical Sciences (TUMS), Tehran, Iran.*; b *Department of Biology, School of sciences, Islamic Azad University, Ashtian Branch, Tehran, Iran.*; c *Department of Biology, School of Sciences, Tehran University of Payame Noor, Tehran, Iran.*

**Keywords:** Micromass culture, Enamel matrix derivative, Neural crest, Teratogen

## Abstract

Enamel matrix derivative Emdogain (EMD) is widely used in periodontal treatment in spite of the fact that its effect on the developing embryo has not been elucidated. The aim of this study was to investigate the teratogenic effect of EMD on the rat embryo neural crest cells. The neural crest is a unique population of cells that migrates from the dorsal neural tube along defined pathways and produces various cell types including the melanocytes, neuronal and glial cells of the sensory, autonomic and enteric nervous system as well as the chromaffin cells of the adrenal gland. These cells have been used extensively for *in-vitro *studies of neurogenesis. Cultured cells by micromass culture method derived from midbrain of six embryos (13 day postcoitum; 34-36 smites) and exposed to various concentrations of EMD for 5 days at 37°C and differentiated foci were counted. Retinoic Acid (20 μg/mL) was used as standard positive control. These cells were stained using Mayer’s hematoxylin which is specific for staining differentiated cell nucleus. Neutral red staining determines cell viability rather than related cell differentiation but is used for normalization of Mayer’s hematoxylin results. At the concentration as low as 8 μg/mL of EMD, no toxic effect on fetal cells was observed and it is suggested that EMD has no teratogenic effect at studied concentrations.

## Introduction

The developing central nervous system is much more susceptible to adverse effects of toxic agents than adults’ CNS system. In fact, among congenital defects, the brain has been introduced as the major target organ of toxicity (1, 2). Moreover, CNS effects are seen most often at lower doses than those required to affect other parts of the body (3-5). Since the use of Enamel matrix derivative (EMD) is being increased every year, there is a great concern about its teratogenic effects in case it is used during the first semester of pregnancy. EMD is a commercially available product isolated from the crowns of six month old pigs. It has been proven to be clinically useful in improving periodontal healing of avulsed and replanted teeth, as it was reported to be effective in treatment of periodontal intra-bony effects (6, 7). Emdogain is a commercial EMD and contains several matrix proteins from the amelogenin family. Several studies have shown that EMD influences. 

the migration, attachment, proliferative capacity and biosynthetic activity of periodontal ligament cells (8-11). Furthermore, it is considered to be effective in enhancing the healing process of replanted teeth and has been recommended as therapeutic agent for the management of avulsed permanent teeth (12-14).

During the critical period of organogenesis, the embryo is especially sensitive to toxic insults of certain drugs and other xenobiotics. The mammalian embryo itself (particularly the rat embryo) can be cultured for a limited period of time to be used as an *in-vitro *teratogenicity assay. Midbrain cells, when grown in high-density cultures, can be differentiated to neurons (15-18).

Rat embryo midbrain cells can be differentiate under particular conditions of culture and produce high density forming neurons (19, 20). Cultures of neural tissue for pharmacological or electrophysiological investigations are normally prepared by dissociating differentiated neurons and glial cells from fetal rat brain (21, 22). One way to optimize the number of high-density cultures obtained from a small quantity of embryonic tissue is using the micromass technique developed by Ahrens, Solursh and Reiter (23). A modification for use with embryonic neural tissue has been described in this paper. Another method which is widely used in teratology is limb bud micromass culture. Limb bud mesenchymal cells, when grown in a micromass culture system, can be differentiated into several of cell types such as cartilage and muscle. Any alteration in expression of the markers which are related to chondrogenesis can be considered as teratogenic characteristics of a xenibiotic. One advantage of this technique is that a homogeneous set of cultures is produced in which differentiation may be followed quantitatively by biochemical or morphometric analysis (24, 25).

## Experimental

*Chemicals*

Mayer’s hematoxylin and neutral red were purchased from Sigma chemicals Co. (St. Louis, Mo, USA). Phosphate buffered saline (PBS), Trypsin, Fetal bovine serum (FBS) and RPMI 1640 medium with L-Glutamine were purchased from Life sciences (Grand Island, NY, USA). EMDs were purchased from Biosera AB, Straumann Emdogain, a company of the straumann Group (SE-205 12 Malmo, Sweden). All other reagents were of the highest available quality and obtained through common commercial sources.

*Animals*

Female Wistar albino rats (250-300 g) housed in a thermostatically-maintained room (22°C, relative humidity 55.5%) with a 12 h light cycle (light from 6:00 am to 6:00 pm), free access to food and tap water and were mated overnight with males of proven fertility (n = 6). The appearance of a vaginal plug on the next morning was considered as an indication of pregnancy. The day of the appearance of the plug was designated as day 0 of gestation. Pregnant females were scarified on day 13 post coitum and the uteri were explanted aseptically and embryos (34-36 somites stage) placed in phosphate buffered saline prewarmed to 37°C under sterile conditions. Midbrain tissue was removed from the embryos postmortem. All the experiments were conducted in accordance with the accepted principles for laboratory animal use and care in TUMS Animal Research Ethics Committee.

*Preparation of neural crest cells*

The midbrain tissues were isolated from a number of embryos, placed and washed in sterile phosphate buffered saline (PBS) followed through placing in a culture medium. Isolation was done by centrifugation (200 g) for 5 min. At the next stage, the tissue fragments incubated in 1x trypsin for 4 min and cells were isolated through centrifugation (300 g for 5 min). The cells were then disaggregated with triturating through a Pasteur pipette. This suspension passed through a sterile 50 μm mesh nylon filter. The resulting single cell suspension was collected and cell viability was determined using trypan blue dye exclusion method. For micromass culture, we used 2 × 10^6^ neural crest cells/mL of stock cell suspension.

*Culture medium*

RPMI 1640 containing 10% fetal bovine serum supplemented with 455 μg/L L-glutamine and 1% Penicillin/Streptomycin (Gibco, UK) and antibiotics (penicillin100 IU/mL, streptomycin 100 μg/mL).

*Micromass culture*

Discrete 20 μL-droplets of the cell suspension (2 × 10^6^ cells/mL) were placed in the center of each well in a 6 well cell culture plate. The prepared dishes were incubated at 37°C in 5% CO_2_, 95% air and 100% humidity for 2 h to allow the cells to adhere. It is important not to disturb the cell during this period. After this time, culture medium was added carefully a final volume of 1 mL.

*Study design*

The 30 mg/mL EMD stock solution was prepared in 1% Trifluoroacetic acid (TFA) and used to make working solutions in culture medium. Neural crest cells were extracted from midbrain and were allowed to grow to confluence for the first 24 h period. The second day of culture, neural crest cells - extracted from midbrain- were treated with various concentrations of EMD (final concentration of 0.5-8 μg/mL of culture medium) for the next 96 h culture period at 37°C.

*Mayer’s hematoxylin staining for cell differentiation assay*

At the end of the 5 day culture period, medium was aspirated from each well of the plate and the wells were washed with PBS solution. Cells were then fixed with 300 μL of 10% v/v aqueous Formaldehyde for 5 min. Fixed cells were rinsed with PBS and then were stained with Mayer’s hematoxylin for 30 sec. The excess amounts of stain were flicked out of the wells and the dishes were gently washed with water. Midbrain cultures are then left to dry in room temperature. The number of stained and differentiated cells in 250 cells was counted in four different parts of microscope object. The percentage of stained cells, compared to the control, was reported. Retinoic acid at concentration of 20 μg/mL (3 × 10-3 M), which can inhabit more than 90% of midbrain differentiation, was used as positive control (24).

*Neutral red staining for cell viability assay*

Three hundred μL of 0.1% neutral red solution was added to each well and incubated for 60 min at 37°C. After this period, the dye was removed and cells were washed with prewarmed PBS. The PBS was replaced with 600 μL acid alcohol (absolute ethanol: 0.1 M citrate buffer, pH = 4.2; 1:1 v/v). The final content was transferred to 96-well plates and was shaken for 20 min at room temperature. The optical density was measured at 540 nm using a plate reader (spectrophotometer).

*Analysis and interpretation of data*

All experiments were repeated at least three times and the results represented as means ± SEM. The effects of the adding concentrations of EMD were assessed using one-way analysis of variance (ANOVA). P- values equal or bellow 0.05 were considered statistically significant difference.

## Results and Discussion

**Figure 1:**
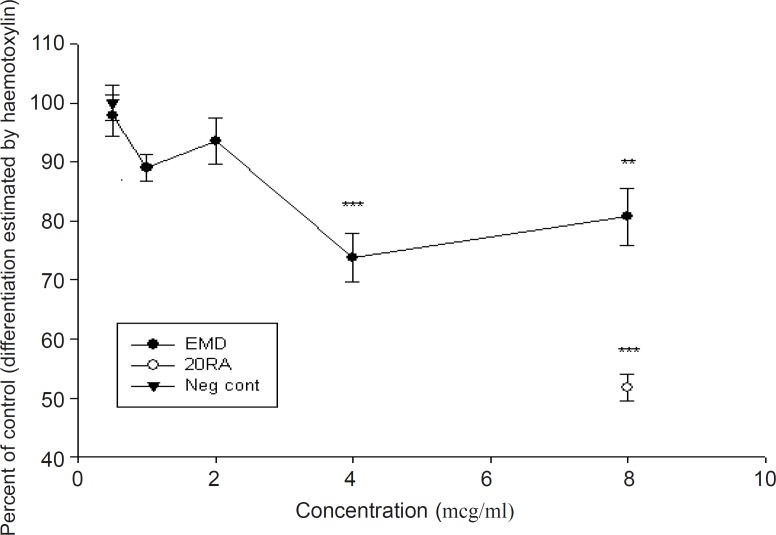
Effect of EMD on differentiation of rat midbrain cells using Mayer's hematoxylin staining method (mean ± SEM). Note that compared to positive control, doses more than 2 μg/mL are biologically significant (p < 0.001).

**Figure 2:**
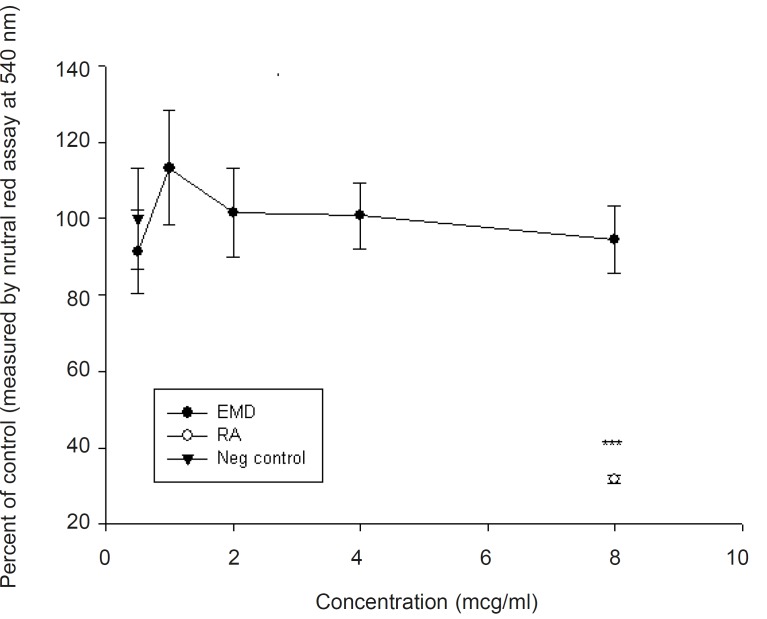
Viability of midbrain cells exposed to different concentrations of EMD using neutral red assay (mean ± SEM). Note that none of the studied doses are statistically significant (p > 0.05).

The results of four experiments, shown in Figure 1, indicate that EMD at concentrations of 0.5-2 μg/mL did not show a biologically significant effect in the number of stained differentiated neural crest cells (p > 0.05), compared to positive control (retinoic acid). At higher doses, EMD significantly reduced differentiation (p < 0.001), although the number of cells which were exposed to EMD do not show any statistically significant differences compared to the negative control group. The results presented in Figure 2 demonstrated that the addition of EMD in different doses directly to the culture medium, do not make any significant alteration in cell viability (p > 0.05). Furthermore, no significant difference was shown between EMD-treated and retinoic acid-treated groups (p > 0.05).

**Figure 3:**
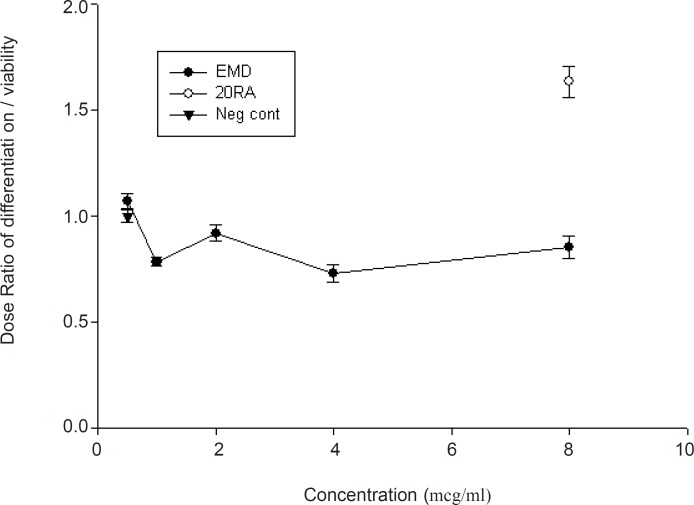
Normalization of midbrain cell differentiation to cell viability following exposure to EMD (mean ± SEM). Note that the ratio between “0.5 or less” and “1.5 or more” is considered biologically teratogen (0.5 ≤ Ratio ≤ 1.5).

Likewise, EMD did not show a statistically significant reduction when the results of neurogenesis were normalized to neutral red absorbance for the viable cell number (Figure 3)

Nowadays, *in-vitro *systems have a worldwide use in toxicity testing. An important scope of problem in toxicology studies consists of developmental neurotoxicity. (26-29). Two widely used methods for studying teratogenic characteristics of xenibiotics are neural crest culture which determines the effect of teratogens on the CNS and limb bud culture which shows the effect of teratogens on chondrogenesis. Micromass culture is a suitable tool to improve the number of differentiated cells in both systems (19, 30-33). Neural cell-based models were used in this study to investigate the teratogenic effect of Emdogain.

When isolated cells from midbrain of developing embryos are cultured at high density, many of the events (movement, communication, division and differentiation), which are most critical to embryogenesis will occur. Therefore, this model, at least in part, resembles *in-vivo *developmental process (2). Developmental toxicity (teratogenicity) of Enamel matrix derivative (EMD) was assessed in this study using modified micromass culture method in which neural crest cells from midbrains were used and criteria were analyzed based on one-way analysis of variance (ANOVA). The differentiation was further evaluated utilizing Mayer’s hematoxylin, a stain which aid in visualization of the differentiated neurons nucleus (2, 16).

The results obtained in this assay indicate that there is no teratogenic effect on fetal cell differentiation when EMD is added to the culture of midbrain cells. The viability was evaluated using neutral red assay method. The results demonstrated that EMD do not produce any significant decrease on embryonic cell viability.

Some previous studies have shown an inhibitory effect of EMD on a variety of epithelial cell types: HT 1080 cell line (34), HeLa cell line (35), epithelial carcinoma cells (36) and oral epithelial cells (37). However, since other studies have shown different proliferative responses to EMD stimulation in diverse cell lines, epithelial cells may also respond in a similar variable manner depending upon their type and embryonic origin.

The majority of viability studies conclude that EMD stimulates the growth, proliferation, and mobility of osteoblasts. EMD also regulates the growth, proliferation and mobility of osteoblastic cell lines and cementoblasts, and their gene expression (38, 39). Besides, EMD plays a role in the differentiation of osteoblasts (40). On the other hand, our results show that EMD did not interfere with embryonic neural stem cells in the case of differentiation.

Taken together, these results indicate that EMD has neither cytotoxic nor teratogenic effect on neural crest cells. These data confirm the results of other studies which show no evidence of toxicity on tongue and dental cells (7, 34-40).
